# Proneness to false memory generation predicts pseudoscientific belief endorsement

**DOI:** 10.1186/s41235-024-00568-4

**Published:** 2024-06-21

**Authors:** Naroa Martínez, Itxaso Barberia, Javier Rodríguez-Ferreiro

**Affiliations:** Grup de Recerca en Cognició i Llenguatge (GRECIL), Departament de Cognició, Desenvolupament i Psicologia de la Educació, Secció de Processos Cognitius, Institut de Neurociències (INUB), Facultat de Psicologia, Universitat de Barcelona (UB), Passeig de La Vall d’Hebron, 171, 08035 Barcelona, Spain

**Keywords:** Unwarranted beliefs, Pseudoscience, False memory, Misinformation

## Abstract

Among cognitive factors that can influence the endorsement of pseudoscientific beliefs, our study focuses on proneness to false memory generation. In this preregistered study, we presented 170 fluent English speakers residing in the USA with a misinformation task aimed at generating false memories. In this task, they first completed an event encoding stage, in which two events were narrated through sequentially presented pictures. One day later, they read a series of sentences relating the same events but which included several inaccurate descriptions aimed at producing a misinformation effect. Finally, we measured the influence of the misinformation manipulation over false memory generation. After completing the misinformation task, participants responded to a questionnaire measuring pseudoscientific beliefs. Our results showed a positive correlation between pseudoscience endorsement and false memory rates, which indicates that the latter might be a key factor influencing susceptibility to pseudoscience. To our knowledge, this is the first study showing a link between the tendency to believe in pseudoscience and variability regarding proneness to develop false memories. Practical implications for the design of new interventions to effectively reduce pseudoscientific beliefs and their negative impact on our society are discussed.

## Introduction

Unwarranted beliefs can be defined as those held in absence of compelling evidence to support them (Lobato et al., 2014). The term encompasses different kinds of beliefs, like those referring to the paranormal (i.e., any phenomenon which, if genuine, would violate the basic principles of science, see Broad, 1949) and to pseudoscience (i.e., any corpus of knowledge presenting itself as scientific but lacking the minimum requirements to be so, see Fasce & Picó, 2019).

Pseudoscientific beliefs related to health are quite prevalent in Western societies. For instance, more than 70% of US adults believe that alternative medicine is a good complement to cancer treatment (American Society of Clinical Oncology, 2019). As for European countries, 86% of French respondents indicate very good or rather good image of alternative medicine (Harris Interactive, 2019), whereas more than 70% of Germans consider that “Alternative medicine is useful for common illness without health consequences” (Zukunftsinstitut, 2017).

This predisposition to pseudotherapy use generates a high cost for society’s economy and health. In this regard, in 2022, the global complementary and alternative medicine market was valued at $117.2 billion and it is expected to reach $694.2 billion by 2030 (a compound annual growth rate of 25.1% from 2023 to 2030; Grand View Research, 2023). In USA, the total out-of-pocket spending on complementary health approaches was $30.2 billion in 2012 (Nahin et al., 2016), and the alternative medicine sector's earnings have been estimated to increase in $8.9 billion in the last 10 years (IBISWorld, 2022). On a personal and social level, people who believe in alternative medicine are more resistant to vaccination (Downey et al., 2010; Jones et al., 2010) and are less likely to use empirically validated medical treatments (Utinans & Ancane, 2014) in favor of pseudoscientific treatments, which might even lead to fatal consequences and an increase of morbidity (Johnson et al., 2018a, 2018b; Lim et al., 2011).

The presence of pseudoscientific beliefs among the general population constitutes a complex phenomenon most certainly rooted in an intricate interplay between social- and individual-level factors. The social component might be especially relevant when referring to pseudoscientific beliefs, as they are likely to proliferate through social interactions such as conversations with relatives, or discussions in online forums or social media. For instance, in a recent survey to the Spanish population, 63.7% of the respondents which had visited a professional with a pseudoscientific approach (e.g., homeopathy, acupuncture, reiki, etc.) reported that they had been informed about it by friends or acquaintances, 22.1% by professionals, and 22.4% through the internet (CIS, 2018). Indeed, exposure to (mis)information regarding pseudoscience (i.e., familiarity) has been pointed out as a key aspect influencing its endorsement (Piejka & Okruszek, 2020), even beyond the preventive influence of disproving information in the case of pseudotherapies (García-Arch et al., 2022; Piejka & Okruszek, 2020).

Nevertheless, it remains unclear why different individuals, all of which have wide access to pseudoscience-related misinformation, present pseudoscientific beliefs to different degrees. In this sense, endorsement of pseudoscience is known to vary along with age (e.g., Torres et al., 2023, though see Fjær et al., 2020), sex (e.g., Fasce et al., 2020; Huete-Pérez et al., 2022; Kemppainen et al., 2018; Majima, 2015), personality (Fasce & Picó, 2019; Furnham, 2007; García-Arch et al., 2022), income (e.g., Eisenberg et al., 1993, 1998; Fjær et al., 2020; Kemppainen et al., 2018), or education level (Astin, 1998; Barnes et al., 2008), among other variables.

Of especial interest to the present study, cognitive factors such as disinclination to analytic cognitive style (Fasce & Picó, 2019; Majima, 2015; Majima et al., 2022) have been hypothesized as possible mediators of the endorsement of pseudoscientific beliefs too. More specifically, cognitive biases such as the jump-to-conclusions bias, i.e., low evidential criteria or a tendency to draw conclusions based on insufficient information, have been reported as possible modulators of the endorsement of these kinds of beliefs (Rodríguez-Ferreiro & Barberia, 2021). In a similar vein, causal illusion, i.e., the false belief that there is a cause-effect relationship between two unrelated events, has been proposed as a basic foundation of pseudoscientific beliefs (Matute et al., 2011). To this regard, individuals developing stronger causal illusions when exposed to non-contingent events in laboratory tasks have also been shown to present stronger endorsement of pseudoscientific beliefs in their daily lives (Torres et al., 2020, 2022).

Following these last studies, the overarching concept driving the present research is that endorsement of pseudoscientific beliefs might be related to variability in the way different individuals process given information. More precisely, here we focus on a possible association between the propensity to endorse pseudoscientific beliefs and the tendency to develop false memories, which refer to “memories for events that never occurred or memories for events that did occur that are grossly distorted” (Lampinen et al., 1998, p. 181). Our proposal is that proneness to false memory could contribute to the development of pseudoscientific beliefs in daily life. In this sense, distortions regarding either content or context of an event could promote the endorsement of different pseudotherapies. For example, falsely remembering that a reiki session relieved your backpain when it did not, misattributing the source of your improvement to a homeopathic pill when you actually took a prescribed med, or erroneously recalling that a health professional recommended Bach flower remedies to you when you actually read about their alleged effectiveness on social media or heard about them from a friend.

The idea that pseudoscience endorsement might be associated in some way with susceptibility to memory distortions has already been tested in previous studies. For instance, in line with results observed regarding the association between political views and false memory promotion (Greene et al., 2021; Murphy et al., 2021), beliefs about vaccination have been shown to influence the generation of false memories for fabricated fake news related with the COVID-19 pandemic, with individuals falsely remembering more fake news congruent with their prior beliefs (Greene et al., 2022; though see King & Greene, 2024 for a lack of association between previous pseudoscientific beliefs and false memories for cancer-related fake news). From a different point of view, Chow et al. (2021) observed that erroneous health beliefs connecting different pseudotherapies and health conditions were frequently associated with the perception of nonzero contingency between those events experienced in one’s daily life. For example, the intensity of the unwarranted belief in herbal remedies as effective treatments for the common cold was associated with the individuals recalling that, among people they knew, the percentage of those improving from their common cold had been higher among the ones taking herbal remedies compared to those not taking them. Even though there exists no known cure for cold, this effect could be due to some individuals having experienced a positive contingency between taking herbal remedies and curing a cold by chance. Nevertheless, as noted by Chow et al. (2021), it is also possible that people better recall those observations that confirm their initial expectations or, we could add, they might even falsely remember them.

These studies can be contextualized in the field of motivated reasoning (Kunda, 1990), as they suggest that pre-existing beliefs might influence the generation of false memories congruent with them. In the present research, nevertheless, we follow a different perspective. Specifically, we propose that, when presented with certain (false) information, a general context-independent proneness to generate false memories could be associated with the development of beliefs congruent with that information. Bringing this idea to the domain of pseudoscientific beliefs, after being exposed to similar degrees of misinformation promoting pseudoscience, a differential tendency to develop false memories could explain, along with other variables, why different individuals end up developing more or less pseudoscience endorsement (see discussion section for alternative interpretations).

Our hypothesis stems from previous results obtained in the study of paranormal beliefs. Meyersburg et al. (2009) observed that individuals presenting (false) memories of past lives were also more prone to develop false memories in the laboratory (see also Wilson & French, 2006, who observed an association between paranormal beliefs and falsely remembering having seen inexistent video footage of dramatic news events). Later on, different authors have also explored a possible association between proneness to false memory generation and magical thinking (i.e., “the belief that events or the behavior of others can be influenced by one’s thoughts, wishes, or rituals”, APA Dictionary of Psychology, n.d.) with mixed results (e.g., Gallo, 2010; Rodríguez-Ferreiro et al., 2020; Saunders et al., 2012). In these studies, false memories were generated by means of the Deese-Roediger-McDermott (DRM) paradigm (Roediger & McDermott, 1995) in which the presentation of a list of words semantically related to an unpresented one are expected to generate false memories of the latter. Nevertheless, although the DRM paradigm is commonly used in false memory research, its relevance for more naturalistic contexts has been criticized (e.g., Pezdek & Lam, 2007; Wade et al., 2007).

In contrast, the misinformation paradigm (e.g., Loftus, 2005) seems more appropriate for our purposes because it involves the construction of an erroneous memory from suggestions and is, hence, more closely aligned with false memories coming from social interaction (Zhu et al., 2013). This paradigm consists of three phases: presentation of a series of pictures describing an original event; presentation of a series of sentences describing the same event but containing misinformation; and a memory test. Studies using the misinformation paradigm show that participants exposed to misinformation after an original event are, to some extent, likely to claim to have seen the misleading details in the original event (e.g., Okado & Stark, 2005). The tendency to develop false memories in this paradigm has been shown to vary between individuals. For instance, with regard to personality, Zhu et al. (2010a) observed that volunteers with lower scores in a novelty seeking scale were more prone to generate misinformation-related false memories. Interestingly, variability with regard to cognitive factors, has also been shown to be associated with proneness to false memory generation, with individuals scoring higher on measures of intelligence, working memory and visual perception, tending to develop less false memories (Zhu et al., 2010b).

All in all, we propose that proneness to generate false memories from external (false) information could be associated with willingness to endorse pseudoscientific claims. To test this hypothesis, we presented volunteers varying in their degree of endorsement of pseudoscientific beliefs with a misinformation task framed in non-pseudoscience-related scenarios. If pseudoscientific beliefs are associated with proneness to memory distortions generated through misinformation, we predict a positive correlation between the rate of the false memories and the scores obtained on the scale measuring pseudoscience endorsement.

## Method

### Participants

We recruited a total of 170 participants (88 females, mean age = 41.2, SD = 13.2), which is double the sample size necessary to detect a medium-size effect (*r* = 0.3) on the critical correlation analysis with a power of 0.80 as indicated by the power analysis performed with G * Power (Faul et al., 2009). Volunteers took part in the study through the online experimentation platform Prolific. All participants were fluent English speakers and U.S. residents.

### Materials and procedure

We implemented the experiment in Qualtrics. We constructed an online false memory test using a misinformation paradigm with the materials provided by Okado and Stark (2005; https://faculty.sites.uci.edu/starklab/false-memory-eyewitness-testimony/), and we included the short version of the Pseudoscience Endorsement Scale (sPES; Torres et al., 2023).

Participants were tested over two sessions separated by a period of 24–26 h. On the first day, the first stage of the false memory task was completed (i.e., the event encoding stage, see below), while on the second day, the second and third stages of the false memory task were completed, followed by the sPES. The first session lasted around 8 min and the second session lasted around 35 min.

#### The false memory task

The false memory task involved four stages: (1) event encoding stage or pictures stage, (2) misinformation or sentences stage, (3) memory test, (4) source monitoring test. Before starting, the participants were told that they were completing a memory task. Following previous studies using this paradigm (Frenda et al., 2014; Zhu et al., 2010a, 2013), we used two different events from the materials developed by Okado and Stark (2005). The first event involved a man breaking into a car and stealing things (henceforth, *car event*), and the second event comprised a repairman who allegedly comes to fix the computer of a research assistant but steals her wallet (henceforth, *computer event*). The order of presentation of these two events was counterbalanced across participants.

*Event encoding stage* Participants were sequentially exposed to images describing the two events. Each event included a set of 50 digital color photographs. Each picture was displayed for around 3,500 ms, with an inter-slide interval of approximately 500 ms. Participants were asked to pay attention to the series of images since they would be asked questions about them later.

*Misinformation stage* Between 24 and 26 h later, the participants read sentences about the same two events that they had observed in the previous stage (the order in which each event was presented to each participant was the same as in the previous stage). For each event, 50 sentences were presented, one after the other. Thirty-eight of those sentences involved accurate written descriptions of the original event (i.e., the information conveyed by the sentences was consistent with the pictures observed in the previous stage). The other 12 (critical) sentences were inaccurate descriptions (i.e., misinformation) of critical pictures of the original event. Following Stark et al. (2010), two alternative versions of each critical picture and sentence were used. For instance, in one of the versions the picture in the first stage showed a man using a *credit card* to open a car door while the sentence in the second stage described a man using a *hanger* to open the car door. In the alternative version, the picture showed the man using the *hanger* while the sentence indicated that he used a *credit card*. Participants observing first the *car event* were exposed to one of these versions (i.e., pictures from tray 2 from the materials provided by Okado and Stark 2005) while participants observing first the *computer event* were exposed to the other (i.e., pictures from tray 1 from the materials provided by Okado and Stark 2005). Like in the first stage, each sentence was displayed for 3500 ms, with an inter-slide interval of 500 ms. Within each event, the sentences were presented in the same order as the previously seen pictures. Participants were instructed to focus on the sentences without warning them that they might encounter inconsistencies.

*Memory test* After a 10–15-min delay (see filler task below), participants were asked to answer 36 (18 per event) forced-choice questions about the information presented in the original event encoding stage (i.e., in the pictures). Twenty-four (12 per event) of these questions (*critical questions*) referred to critical pictures (those for which the subsequent sentences had included misinformation), and the remaining 12 (6 per event) questions were control questions related to non-critical pictures (pictures for which the subsequent sentences had presented consistent information). We highlighted in the instructions that they should answer based on what they had seen in the pictures. Each question included three possible answers. In the critical questions, one of the alternatives matched the information presented in the pictures of the original event encoding stage (*correct* response), another alternative was consistent with the misleading information given in the sentences (*misinformed* response), and a third alternative referred to an aspect neither seen in the pictures nor read in the sentences (*new* response). In the control questions, one of the options corresponded to the correct response, and the other two options involved new responses. The order of presentation of the questions and the answer alternatives were randomized within each event and across subjects, but respecting the order of the events observed by each participant on the first and second stages. Following Zhu et al. (2010a), the answers to this test allowed us to calculate false memory rate, our main dependent variable, which is the proportion of misinformed responses on the critical questions presented in the memory test. This rate represents the tendency of the participants to incorporate misinformation from the sentences in their responses to critical questions about the pictures. Additionally, responses to this test allowed to calculate a correct memory index (as in Frenda et al., 2014), as the proportion of correct responses for the control items.

*Source monitoring test* For consistency with previous studies on the topic of false memories generated through misinformation, we also gathered *robust* false memory scores (i.e., rates of false memories mistakenly attributed to the legitimate source). Immediately after the memory test, participants completed the source monitoring test, in which they were again presented with all the critical questions of the memory test and they were asked to indicate the source that they had used to answer the memory test. The four alternatives given were: "I saw it in the pictures only," "I read it in the sentences only," "I both saw it in the pictures and read it in the sentences," and “I guessed.” These response options were adapted from Zhu et al. (2010a), except that we changed the original alternative “saw it in both and they were the same” to “I both saw it in the pictures and read it in the sentences”, and we also removed the alternative “saw it in both and they conflicted with each other” because we considered it to be ambiguous. Therefore, our source monitoring test included 4 response alternatives instead of 5.

Following Zhu et al. (2010a), the answers to these tests allowed us to calculate *robust* false memory scores,^1^ which are calculated as the proportion of cases, among those in which the participant chose the misinformed alternative in the previous memory test, in which they further answered "I saw it in the pictures only” or "I saw it in both” on the present source memory test.

#### Pseudoscience endorsement scale

The short version of the Pseudoscience Endorsement Scale (sPES; Torres et al., 2023) consists of 12 statements aimed to assess the endorsement of pseudoscientific beliefs (e.g. “Natural remedies, such as Bach flower remedies, help overcome emotional imbalances.”). Responses are provided on a Likert-like scale ranging from 1 (i.e., “Strongly disagree”) to 7 (i.e., “Strongly agree”). The final sPES score is obtained by averaging the ratings given to the different statements. This questionnaire has obtained excellent internal consistency in previous studies (McDonald’s *ω* = 0.90, see Torres et al., 2023). Scores obtained on this scale have been shown to positively correlate with variables such as age, right-wing ideology and receptivity to what is known in the literature as “pseudo-profound *bullshit*” (i.e., obscure and vague language designed to impress but lacking actual meaning, Pennycook et al., 2015, often used to support pseudoscientific claims), and to negatively correlate with cognitive reflection and science literacy (Torres et al., 2023). Interestingly, positive associations have also been observed between PES scores and both intensity of causal illusions (Torres et al., 2020, 2022) and proneness to overadministration of ineffective treatments in a laboratory task (Torres et al., 2022, see filler task in the present study).

#### Filler task

As a filler task (introduced between the second and the third stage of the false memory task), we adapted a standard contingency judgment task that assesses the tendency to develop causal illusions (e.g., Barberia et al., 2019; see Matute et al., 2015, for a review). Responses to this task, which have been shown to correlate with pseudoscientific belief endorsement (Torres et al., 2020, 2022), allowed us to explore a possible relation between proneness to false memory generation and the development of causal illusions. Participants had to judge the effectiveness of a experimental substance to cure headache. A sequence of trials corresponding to medical records of patients suffering from headaches was presented to them on a computer screen. Specifically, each record showed whether a given patient had taken the substance or not and they were asked whether they believed that the headache would disappear or not within the next two hours (by giving a yes/no response). Then, they received feedback indicating whether the patient’s headache disappeared or not. Out of 48 patients presented, 36 took the substance, whereby the headache disappeared in 27 cases and persisted in 9. The remaining 12 patients did not take the substance, from which the headache disappeared in 9 cases and persisted in 3. Therefore, the substance was useless for treating headache, since the probability that the headache would disappear after taking the substance was the same as the probability that the headache would disappear after not taking the substance, i.e., P(Cure|Substance) = P(Cure|¬Substance) = 0.75, making the contingency 0. The order of the different types of trials was randomized for each participant. After all medical records had been presented, the participants were asked to judge the effectiveness of the substance as a cure for headache on a scale between 0 and 100 where 0 meant "not effective" at all and 100 meant "fully effective." These effectiveness ratings indicate the extent to which a causal illusion has been developed.

## Results

The hypothesis, protocols, sample size, and analysis plan were preregistered in AsPredicted [https://aspredicted.org/sw3ki.pdf], and the resulting dataset is available at OSF [https://osf.io/z69yk/]. Data were analyzed with Jamovi (version 2.4.1). We conducted Kendall’s tau for all correlation analyses since the Shapiro–Wilk test showed that the mean false memory rate, *robust* false memory score, correct memory index, sPES scores, and effectiveness ratings in the filler task did not follow a normal distribution, all *ps* ≤ 0.004.

Note that, as indicated in our preregistration, we consider the overall false memory rate to be the main dependent variable measuring false memory in our study, and our preregistered hypothesis states that this value should positively correlate with the level of endorsement of pseudoscience (sPES scores). The other indexes derived from the false memory task (i.e., *robust* false memory score and correct memory index) were considered secondary and the analysis of their relationship with pseudoscientific beliefs was planned as exploratory.

Analyses of the false memory rates, mean = 0.44, SD = 0.18, 95% CI [0.41, 0.47], showed acceptable reliability, *ω* = 0.74. However, note that, when analyzing each event separately, we observed a value of *ω* = 0.71 for the *car event* but a poorer *ω* = 0.50 for the computer event. An inspection of the item-test correlation of this later event revealed that one of the items was negatively associated with the total false memory rate (*r* = − 0.31). Further inspection of this item showed that its formulation was problematic^2^ and its removal increased reliability for this event up to *ω* = 0.58. Therefore, we opted to eliminate this item from all of the subsequent analyses (new overall mean false memory rate = 0.45, SD = 0.19, 95% CI [0.42, 0.48], *ω* = 0.77, after eliminating this item). Correlational findings including this item did not differ from the findings presented here.

With regard to the pseudoscientific endorsement scale, sPES scores showed excellent consistency in our sample (*ω* = 0.92). Participants obtained a mean score of 3.90, SD = 1.20, 95% CI [3.72, 4.08], on a scale from 1 to 7, where higher scores correspond to stronger pseudoscientific beliefs.

**Fig. 1:**
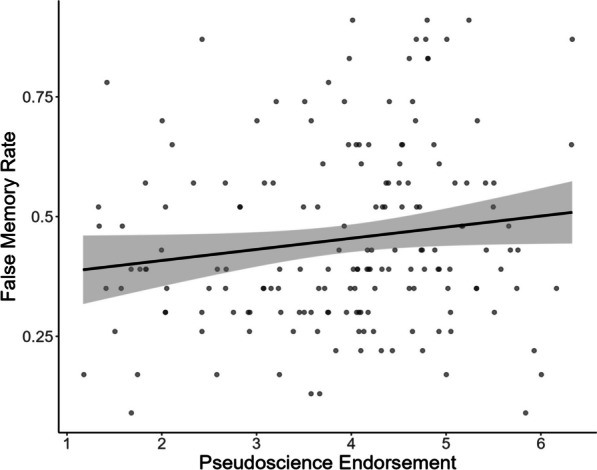
Correlation between false memory rates and scores on the pseudoscience endorsement scale. The gray area indicates 95% confidence intervals

Crucially, false memory rate was positively correlated with endorsement of pseudoscientific beliefs, *r*τ = 0.11, *p* = 0.036 (see Fig. 1). Although not contemplated in our preregistered analysis, given the difference in terms of reliability between the two events, we decided to conduct separate correlational analyses for each of them, in order to ascertain whether problems with the task could be obscuring the results, especially after having noted the reliability problems of one of them. Interestingly, the analyses showed that false memory rate was positively correlated with the endorsement of pseudoscientific beliefs in the (more reliable) *car event*, *r*τ = 0.14, *p* = 0.012, but not in the (less reliable) *computer event*, *r*τ = 0.08, *p* = 0.149.

Finally, exploratory analyses involving the relationship between either *robust* false memory score,^3^ mean = 0.49, SD = 0.26, 95% CI [0.45, 0.53], or the correct memory index, mean = 0.61, SD = 0.16, 95% CI [0.58, 0.63], and pseudoscientific beliefs, showed no significant associations, *r*τ = 0.05, *p* = 0.36, and *r*τ = − 0.02, *p* = 0.77, for *robust* false memory score and correct memory index, respectively.

Regarding the results of the filler task, although it was not part of the main goal of the study, we ran a correlational analysis which returned a positive association between the intensity of the causal illusion developed in the contingency learning task and the level of endorsement of pseudoscientific beliefs, *r*τ = 0.134, *p* = 0.011, but not with regard to performance on the false memory task, *r*τ = 0.001, *p* = 0.979.

## Discussion

Our study aimed to examine whether variability with regard to the tendency to develop false memories from misinformation contributes to the endorsement of pseudoscientific beliefs. Our hypothesis is that individuals more prone to develop false memories would also be at a higher risk of developing pseudoscientific beliefs, as these distorted memories would facilitate them to hold beliefs at odds with the actual observed evidence. Consistent with this hypothesis, we found a significant positive correlation between the intensity of a range of pseudoscientific beliefs held by our participants and their rate of misinformed responses in a domain-independent false memory task.

Previous literature has shown that different cognitive factors modulate the tendency to endorse pseudoscientific beliefs, such as predisposition to analytic thinking (Fasce & Picó, 2019; Majima et al., 2022) as well as cognitive biases related to jumping to conclusions (Rodríguez-Ferreiro & Barberia, 2021) and causal illusion (Torres et al., 2020, 2022). Although these results already suggest that the tendency to believe in pseudoscience may be related to variability in cognitive processing, to our knowledge, this is the first study showing a link with proneness to develop false memories. Nevertheless, the observed effect size is not large, thus aligning with the idea that pseudoscientific belief endorsement is influenced by a multiplicity of factors. To this respect, proneness to false memory generation appears to be independent from the tendency to develop a causal illusion, as suggested by the lack of correlation between the measures of these two constructs in our results, despite both of them being associated with the presence of pseudoscientific beliefs. This observation points to a differential role of cognitive processes responsible for integrating given (mis)information as opposed to those involved in learning from experience.

Our results have both theoretical and practical implications. On the one hand, they extend previous findings on the cognitive aspects that may be modulating pseudoscientific beliefs by providing evidence of an association with proneness to false memory. The correlational nature of our data does not allow us to establish a reliable cause-effect relationship between these two phenomena, nor to determine the direction of the relation in case it is indeed causal. In this sense, our hypothesis is that false memories could lead to pseudoscience endorsement, which is not incompatible with the possibility that individuals who already believe in the efficacy of a given pseudotherapy, then generate false memories to support that belief (see Greene et al., 2022). In fact, both could be true so that beliefs and false memories feed back on each other and are mutually reinforced.

Still, in our view, the fact that the effect was observed with a false memory task conceptually unrelated to pseudoscientific claims indicates that the association does not rely on prior beliefs or expectations with regard to any specific topic, thus giving support to our original proposal. With regard to this, note that future researchers should carefully consider which misinformation events to incorporate in their experimental design, taking into account the differences in reliability observed in the present work, with the *car event* obtaining far better reliability values than the *computer event*. In any case, experimental generation of false memories regarding pseudo-treatments and subsequent assessment of their influence over pseudoscientific belief generation or pseudoscience-associated behaviors could be carried in the future to further corroborate our hypothesis.

Although the present research does not allow to deepen into the cognitive mechanism underlying the observed effect, it is worth contextualizing our results with regard to the main hypothesis usually applied to the study of false memory generation: the fuzzy trace theory (Reyna & Brainerd, 1995) and the source monitoring framework (Johnson et al., 1993). On the one hand, according to the fuzzy trace theory (Reyna & Brainerd, 1995), memory entails parallel encoding, storing and retrieving of verbatim and gist representations. The former refers to specific details of a sentence or situation, whereas the latter refer to its general meaning. Interestingly, gist memory has been shown to be more influential than verbatim over decision making (see Blalock & Reyna, 2016, for a review) and, as we have already mentioned in the introduction, individuals tend to falsely remember verbatim details congruent with their beliefs or gist (Greene et al., 2022, see Langdon et al., 2024 for a review and commentary). From this perspective, false memories generated in our task rely on verbatim traces as participants were asked about specific details of the presented situations, so the association between this kind of false memories and beliefs do not necessarily align with the results of previous studies. Note, however, that if a complementary interplay between gist and verbatim memory is assumed, an individual with a tendency to focus on gist information might also tend to ignore verbatim details, thus leading to more false memories. In any case, it is worth remembering that our novel hypothesis entails general context-independent proneness to false memory generation influencing belief endorsement (and not beliefs influencing the generation of congruent false memories). On the other hand, in the context of the source monitoring framework (Johnson et al., 1993) our results could be interpreted as increased difficulties to correctly attribute the observed information to its actual source for individuals more strongly endorsing pseudoscientific beliefs. From this perspective, the observed errors would indicate failures in either heuristic (e.g., based on perceptual or temporal cues) or deliberate (e.g., systematic assessment of plausibility) judgments of truthfulness (see Murphy et al., 2021, for similar interpretations). For instance, taking up one of the examples presented in the introduction, believers would be more susceptible than sceptics to mistakenly attribute a recommendation read in social media to a health professional.

As for the practical implications, this research may serve as a basis for the design and implementation of strategies aimed at reducing the presence of pseudoscientific beliefs in the population. According to our results, and assuming that proneness to false memory has a causal role over acceptance of pseudoscience, we could expect that alerting about the fallibility of our memory could lead to greater resistance to the misinformation effect and, in turn, less endorsement of pseudoscientific beliefs. Additionally, and following Larrick (2004) with regard to the necessity to complement “training in bias” (i.e., educating regarding the existence of specific cognitive biases) with “training in rules” (i.e., providing strategies to overcome them or guidance on how to optimally make decisions), a successful intervention might also need to include the presentation of clear information about the efficacy of the products and/or treatments, which should serve as a basis to modify beliefs established resulting from erroneous memories. In this sense, we strongly recommend the implementation of empirically supported methods to address pseudoscientific beliefs, such as the motivational interview (Gagneur, 2020) or the empathetic motivational interview (Holford et al., 2024), which emphasize the use of respectful and empathetic approaches to avoid backfire effects.

## Data Availability

The raw data generated and/or analyzed during the current study are available at the Open Science Framework: https://osf.io/z69yk/?view_only=1e5774aee5224651a3541892dd0a914f

## Footnotes

1 Although the *robust* memory score has been calculated in previous studies applying the misinformation paradigm (e.g., Zhu et al., 2010a), we deem it potentially confusing for the participants, so in the present research we focused our hypothesis on the general false memory rate, which we consider to be a purer measure of false memory generation (see preregistration).

2 We refer to the question “What did the repairman specifically put into his tool bag?” of the computer event from the original materials, which included the response options “A stack of CDs”, “A mouse” and “Printer paper”. We consider that the question is invalid to capture false memory because the sentence from the misinformation stage that should allegedly produce a misinformation effect actually corresponds correctly to the picture shown in the event encoding stage (i.e., when the picture showed the repairman introducing a mouse in his tool bag, the sentence would read “He put the computer mouse in his tool bag” and when the picture showed him introducing a stack of CDs, the sentence would read “He put the stack of CDs in his tool bag”).

3 Note that the adaptations we conducted in the procedure to assess this score make our results not directly comparable with those of previous studies.
